# Roles of neutrophil gelatinase-associated lipocalin (NGAL) in human cancer

**DOI:** 10.18632/oncotarget.1738

**Published:** 2014-02-01

**Authors:** Saverio Candido, Roberta Maestro, Jerry Polesel, Alessia Catania, Francesca Maira, Santo S. Signorelli, James A. McCubrey, Massimo Libra

**Affiliations:** ^1^ Department of Bio-medical Sciences, Section of Pathology & Oncology, Laboratory of Translational Oncology & Functional Genomics, University of Catania, Catania, (Italy);; ^2^ Epidemiology and Statistic Unit, CRO National Cancer Institute, Aviano, (Italy);; ^3^ Unit of Experimental Oncology 1, CRO National Cancer Institute, Aviano, (Italy);; ^4^ Department of Medical and Pediatric Sciences, Medical Angiology Unit; University of Catania, Catania (Italy);; ^5^ Brody School of Medicine at East Carolina University, Department of Microbiology & Immunology, Greenville, NC, (USA);

**Keywords:** NGAL, LCN2, Lipocalin 2, mRNA expression, cancer, biomarker, prognostic factor, metastasis, protein expression

## Abstract

Cancer remains one of the major cause of death in the Western world. Although, it has been demonstrated that new therapies can improve the outcome of cancer patients, still many patients relapse after treatment. Therefore, there is a need to identify novel factors involved in cancer development and/or progression. Recently, neutrophil gelatinase-associated lipocalin (NGAL) has been suggested as a key player in different cancer types. Its oncogenic effect may be related to the complex NGAL/MMP-9. In the present study, NGAL was analyzed at both transcript and protein levels in different cancer types by analysing 38 public available microarray datasets and the Human Protein Atlas tool.

NGAL transcripts were significantly higher in the majority of solid tumors compared to the relative normal tissues for every dataset analyzed. Furthermore, concordance of NGAL at both mRNA and protein levels was observed for 6 cancer types including bladder, colorectal, liver, lung, ovarian, and pancreatic. All metastatic tumors showed a decrease of NGAL expression when compared to matched primary lesions.

According to these results, NGAL is a candidate marker for tumor growth in a fraction of solid tumors. Further investigations are required to elucidate the function of NGAL in tumor development and metastatic processes.

## INTRODUCTION

Metastatic spreading is the major cause of death in cancer patients. Several molecules have been shown to contribute to tumor invasion and spreading. In this manuscript the roles of neutrophil gelatinase-associated lipocalin (NGAL) in cancer development and progression are described.

**Figure 1:**
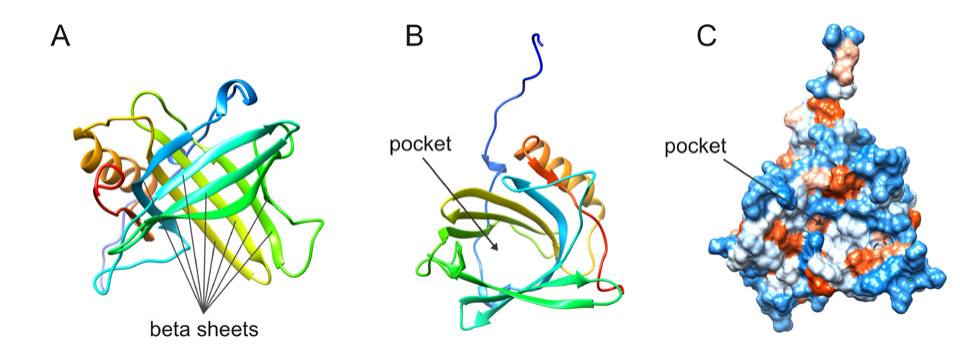
Highly conserved lipocalin crystal structures consist of a single eight-stranded continuously hydrogen-bonded antiparallel β-barrel (A) delineating a calyx shape, which represents the internal ligand-binding site (B). Hydrophobicity surface (C) Images were created from the RCSB PDB database (http://www.rcsb.org) (ID: 1NGL) using the UCSF Chimera package UFCS Chimera package that is developed by the Resource for Biocomputing, Visualization, and Informatics at the University of California, San Francisco (supported by NIGMS P41-GM103311). Ref: The solution structure and dynamics of human neutrophil gelatinase-associated lipocalin by Coles M, et al. J Mol Biol. 1999; 289: 139-57.

Members of the lipocalin protein family are characterized by their ability to bind small hydrophobic molecules (such as prostaglandins, retinoids, arachidonic acid, hormones and fatty acids). They often bind to specific cell-surface receptors and form macromolecular complexes. Highly conserved lipocalin crystal structures consist of a single eight-stranded continuously hydrogen-bonded antiparallel β-barrel delineating a calyx shape, which represents the internal ligand-binding site (Figure 1). Members of the lipocalin family, in the past classified exclusively as transport proteins, have now been described to carry out a variety of different functions. Some of these functions include: retinol transport, cryptic coloration, olfaction, pheromone transport, and the enzymatic synthesis of prostaglandins, moreover the lipocalins are also involved in the regulation of the immunoresponse and the mediation of cell homoeostasis [1].

NGAL, also called lipocalin 2 siderocalin and 24p3, was identified in several forms: a monomer (25-kDa), a disulfide-linked homodimer (46-kDa), and a disulfide-linked heterodimer with human neutrophil gelatinase B (135-kDa) [2]. NGAL has several functions. In early studies NGAL was described as a factor of innate immune system. NGAL is released by neutrophils at sites of infection and inflammation to sequester bacterial ferric siderophores, participating in the antibacterial iron-depletion strategy of innate immune system [3]. Subsequently, it was shown that NGAL is responsible for iron delivery to the cytoplasm where it is accumulated and activates or represses iron-responsive genes. Iron unloading depends on the cycling of NGAL through acidic endosomes [4]. In contrast, Devireddy LR *et al* have shown that NGAL is also involved in apoptosis-dependent deprivation of trophic factors. Apo-NGAL, after binding to its putative receptor, 24p3R, is internalized and associates with an intracellular siderophore, transferring chelated iron to the extracellular medium, thereby reducing intracellular iron concentration which leads to the expression of the pro-apoptotic protein Bim, leading to the induction of apoptosis [5].

NGAL was originally identified as a protein covalently associated with 92-kDa gelatinase/MMP9 from human activated neutrophils [2]. NGAL is expressed in many other types of cells in response to various injuries, especially in kidney diseases. Serum NGAL levels correlate clearly with the severity of renal injury, reflecting the degree of tissue damage. For this reason, NGAL may become one of the most promising next-generation biomarkers in clinical nephrology and as well as other diseases and pathological states [6].

**Figure 2:**
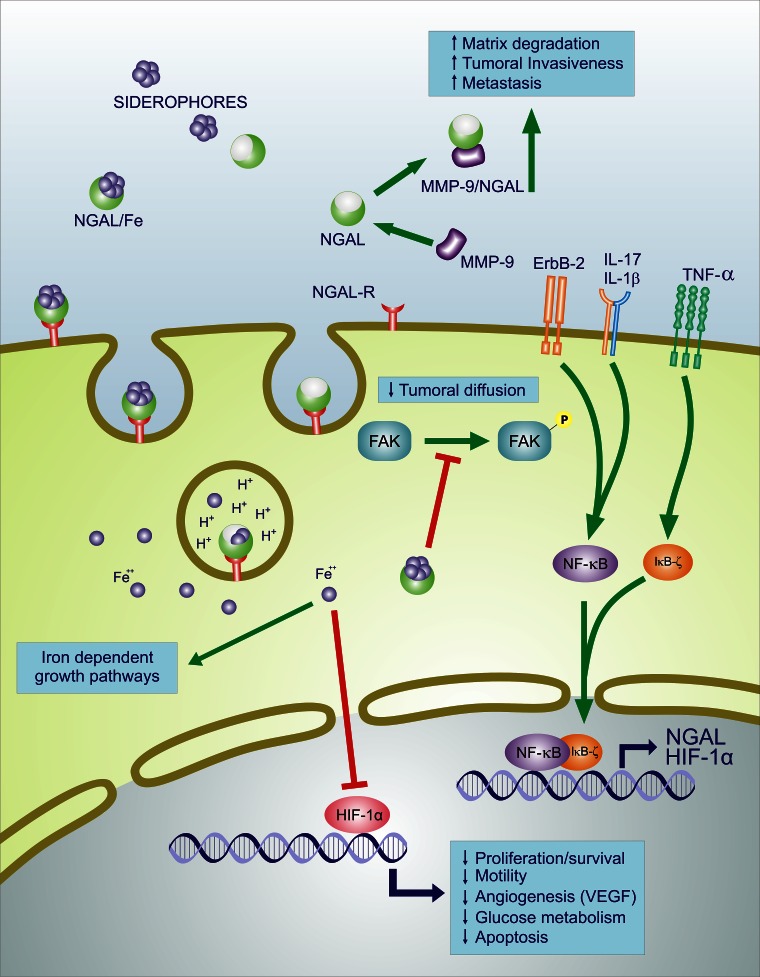
Effects of NGAL on survival, motility, angiogenic, apoptotic and glucose metabolism NGAL: neutrophil gelatinase-associated lipocalin; MMP9: matrix metalloproteinase 9; NGAL-R: NGAL receptor; ErbB-2: v-Erb-B2 Avian Erythroblastic Leukemia Viral Oncogene Homolog = HER2; TNF-α: Tumor Necrosis Factor Alpha; IL-17: interleukin-17; IL-1β: interleukin-1 beta; FAK: focal adhesion kinase; NF-κB: nuclear factor kappa B cells; IκBζ: Inhibitor of NF-κB zeta subunit; HIF-1α: hypoxia inducible factor 1 alpha; VEGF: vascular endothelial growth factor. Fe++: Ferrous iron; Green lines with arrows indicate activation of pathway, red lines with blocked ends indicate inhibition of pathway, blue lines indicate transcriptional activation and additional events.

NGAL is up-regulated by IL-1 beta, but not by TNF-alpha, in type II pneumocyte-derived cell line through the induction of the NF-kB pathway [7]. IL-1 beta selectivity in inducing NGAL is due to the synthesis of IkB-zeta, a NF-kB-binding cofactor, elicited specifically by IL-1beta stimulation which is required for transcriptional activation of NGAL [8]. Stimulation with TNF-alpha in the presence of IL-17, which stabilizes the IkB-zeta transcript, is able to induce NGAL expression by IkB-zeta protein binding to NF-kB on the NGAL promoter [9]. It has been also demonstrated that activation of the NF-kB pathway is associated with up-regulation of NGAL-ErbB2-mediated signaling [10] (Figure 2).

NGAL's ability to combine in a dimeric complex with MMP-9, results in a protective action of MMP-9 from its auto-degradation and consequently results in a higher gelatinolytic action of MMP-9 on extracellular matrix [11]. By this function, it has been shown that NGAL may promote cancer development in a variety of different cancer types [12-15] (Figure 2). Conversely, anticancer activities of NGAL have been demonstrated by its ability to inhibit the pro-neoplastic factor HIF-1a, the synthesis of HIF-1a-dependent VEGF [16,17], and phosphorylation of FAK kinase [17], as shown in colon [18], ovarian [19] and pancreatic [17] cancers.

Further evidences indicate that NGAL plays key roles in the inflammation and in the regulation of cell growth and adhesion in both normal and tumor tissues [20-23].

In the present study, NGAL transcript levels and its potential clinical implications in different cancer types were examined by bioinformatic approaches. NGAL transcript levels were explored in different cancer types by analysing public available microarray datasets. Further evaluation of NGAL protein expression were performed by analyzing the Human Protein Atlas. According to both the results of the present analyses and previous published data, NGAL potential clinical implications are also discussed.

## RESULTS

### mRNA expression of NGAL in different tumor types

Gene expression patterns of NGAL mRNAs present in different tumor types were obtained from several datasets (Table 1). Significant differences between tumor tissues and relative normal counterparts for each cancer type are reported in Table 1. This analysis showed that NGAL transcript levels were significantly higher in the majority of solid tumors compared to the relative normal tissues for every dataset analyzed. While, lower levels of NGAL were observed in each dataset of cervical cancer, esophageal cancer, head and neck cancer and in haematological malignancies. In Table 2 are presented the significant differences of NGAL transcript levels observed in metastatic tissues compared to those of the relative primary tumor. The results showed that in all dataset analyzed the levels were significantly lower in the metastatic tissues than in primary tumors from 5 different tumor types, including colorectal, kidney, melanoma, ovarian and prostate (Table 2).

**Table 1:** Gene expression patterns of NGAL in different cancer types from 29 datasets

Cancer Type	# of samples		Fold Change (≤-2) or (≥2)	p < 0.01 (T-test)	Dataset		
	Cancer	Normal			Author [Ref.]	Year	Platform
Solid tumor							
Bladder	109	48	4.13	2.75E-05	Sanchez-Carbayo M [86]	2006	U133A
							
Cervix	32^a^	24	-3.21	4.06E-04	Scotto L [87]	2008	U133A
Colon	95	5	2.62	1.23E-02	Kaiser S [88]	2007	U133 Plus 2.0
	81	24	4.15	7.04E-08	Skrzypczak M [89]	2010	U133 Plus 2.0
	70	12	4.78	7.06E-06	Hong Y [90]	2010	U133 Plus 2.0
Esophagus	17^b^	17	-5.41	9.27E-05	Hu N [91]	2010	U133A
	53^b^	53	-2.92	1.05E-06	Su H [92]	2011	U133A/B
Head and Neck	6^d^	4	-18.58	7.33E-03	Schlingemann J [93]	2005	U133A
	31^c^	10	-12.44	3.60E-09	Sengupta S [94]	2006	U133 Plus 2.0
kidney	51^e^	5	4.15	4.98E-05	Yusenko MV [95]	2009	U133 Plus 2.0
Liver	35	10	8.66	2.32E-05	Wurmbach E [96]	2007	U133 Plus 2.0
	22	21	3.48	5.14E-04	Roessler S (1) [97]	2010	U133 Plus 2.0
	225	220	2.94	6.86E-22	Roessler S (2) [97]	2010	HT U133A
Lung	30^f^	30	2.79	1.72E-03	Su LJ [98]	2007	U133A
	58^f^	49	2.28	2.64E-06	Landi MT [99]	2008	U133A
	226^f^	20	3.707	1.53E-7	Okayama H [100]	2012	U133 Plus 2.0
Ovary	185^g^	10	5.84	1.54E-06	Bonome T [101]	2008	U133A
	99^g^	4	3.03	6.98E-04	Hendrix ND [102]	2006	U133A
Pancreas	36^h^	16	14.05	5.15E-06	Pei H [103]	2009	U133 Plus 2.0
	11^h^	6	10.00	9.03E-05	Segara D [104]	2005	U133A
	39^i^	39	7.70	1.64E-10	Badea L [105]	2008	U133 Plus 2.0
Thyroid	9^j^	9	3.77	9.76E-4	He H [106]	2005	U133 Plus 2.0
	14^j^	4	2.33	0.001	Vasko V [107]	2007	U133 Plus 2.0
	26^j^	4	2.05	9.34E-7	Giordano TJ [108]	2006	U133A
Hematologic tumor							
ALL	750	74	-11.77	7.57E-152	Haferlach T [109]	2010	U133 Plus 2.0
AML	542	74	-16.94	2.32E-165	Haferlach T [109]	2010	U133 Plus 2.0
	285	8	-4.91	5.00E-03	Valk PJ [110]	2004	U133A
CLL	448	74	-42.98	7.73E-194	Haferlach T [109]	2010	U133 Plus 2.0
Myeloma	9k	5	-3.90	0.002	Agnelli L [111]	2009	U133A

Legend: Solid tumor: Cervix: ^a^Cervical Squamous Cell Carcinoma. Esophagus: ^b^Esophageal Scquamous Cell Carcinoma; Head and Neck: ^c^Nasopharyngeal Carcinoma; ^d^Squamous Cell Carcinoma. Kidney: ^e^Renal Carcinoma; Liver: (1), dataset 1; (2) dataset 2; Lung: ^f^Lung adenocarcinoma; Ovary: ^g^Ovarian Carcinoma; Pancreas: ^h^Pancreatic Carcinoma; ^i^Pancreatic Ductal Adenocarcinoma; Thyroid: ^j^Thyroid Gland Papillary Carcinoma

Hematologic tumor: ALL, Acute Lymphoblastic Leukemia; AML, Acute Myeloid Leukemia; CLL, Chronic Lymphocytic Leukemia.

Myeloma: kPlasma Cell Leukemia.

**Table 2:** NGAL transcripts in metastatic tissues compared to the relative primary tumor

Cancer Type	# of samples		FC (≤ -1.5) or (≥ 1.5)	p < 0,05 (T-test)	Data set		
	metastasis	primary			Author [Ref.]	Year	Platform
Colorectal	43	330	-3.191	1.56E-06	Bittner M[a]	2005	U133 Plus 2.0
	27	56	-5.437	1.04E-06	Tsuji S [112]	2012	U133 Plus 2.0
Kidney	60	9	-1.960	4.00E-03	Jones J [113]	2005	U133A
Melanoma	40	16	-2.475	3.00E-03	Riker AI [114]	2007	U133 Plus 2.0
	52	31	-4.849	1.12E-08	Xu L [115]	2008	U133A
Ovarian	75	166	-1.999	2.00E-03	Bittner M[a]	2005	U133 Plus 2.0
	16	74	-1.537	4.30E-03	Anglesio MS [116]	2008	U133 Plus 2.0
Prostate	5	27	-1.578	2.60E-02	Vanaja DK [117]	2003	U133A/B
	6	7	-5.735	7.76E-04	Varambally S [118]	2005	U133 Plus 2.0

[a] GEO Series GSE2109; FC, Fold change

**Figure 3:**
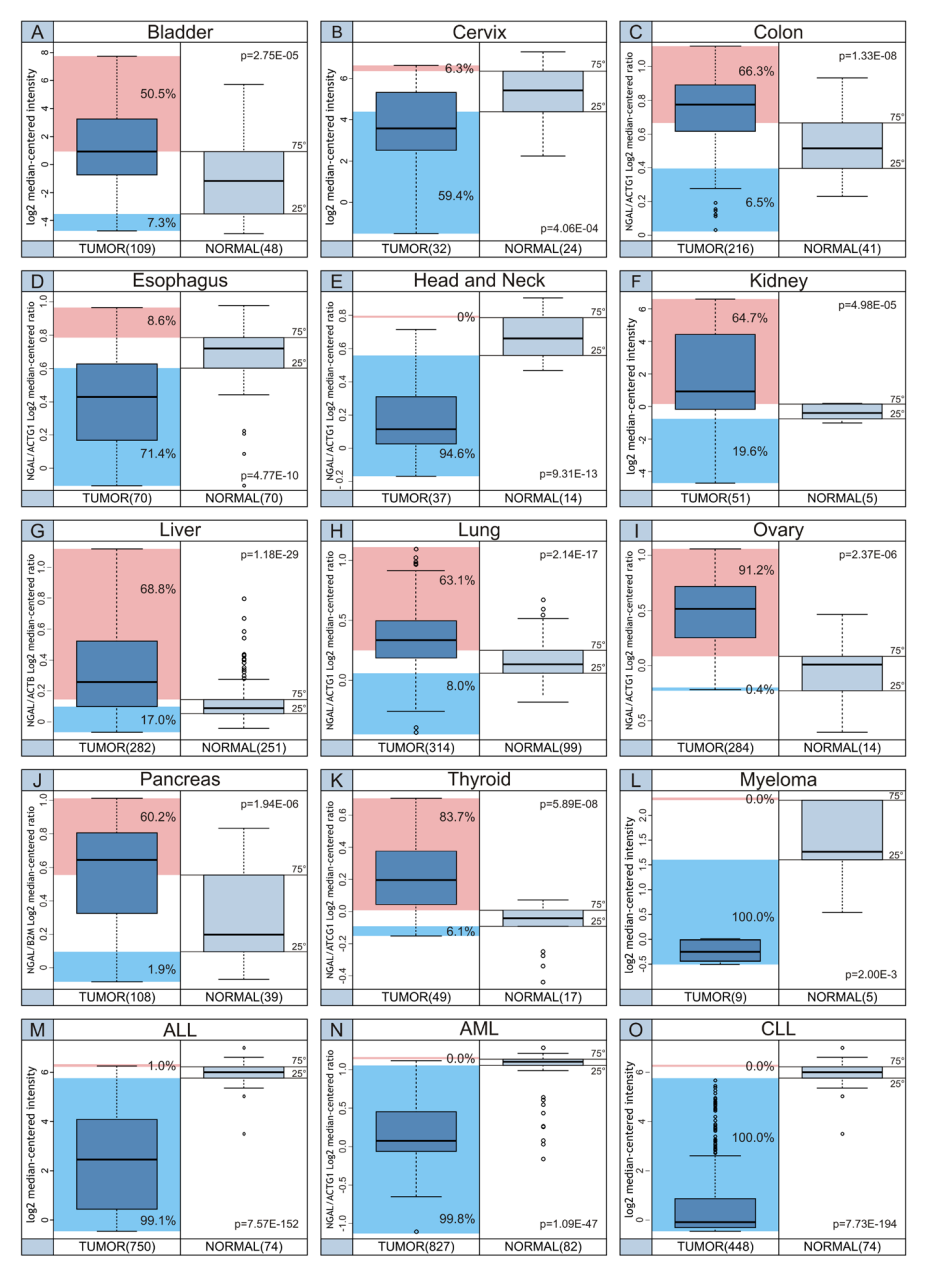
Distribution of NGAL transcript levels among cancer cases and normal samples The percentage of tumor cases, indicated for each tumor setting, shows NGAL transcript levels below the 25^th^ percentile (Cyan box) and above the 75^th^ percentile (Magenta box) of the “normal” samples.

In Figure 3 the distribution of NGAL transcript levels among cancer cases and normal samples is shown. The percentage of tumor cases showing NGAL transcript levels below the 25^th^ percentile and above the 75^th^ percentile of the “normal” samples is also reported.

### Protein expression of NGAL in different tumor types

**Figure 4:**
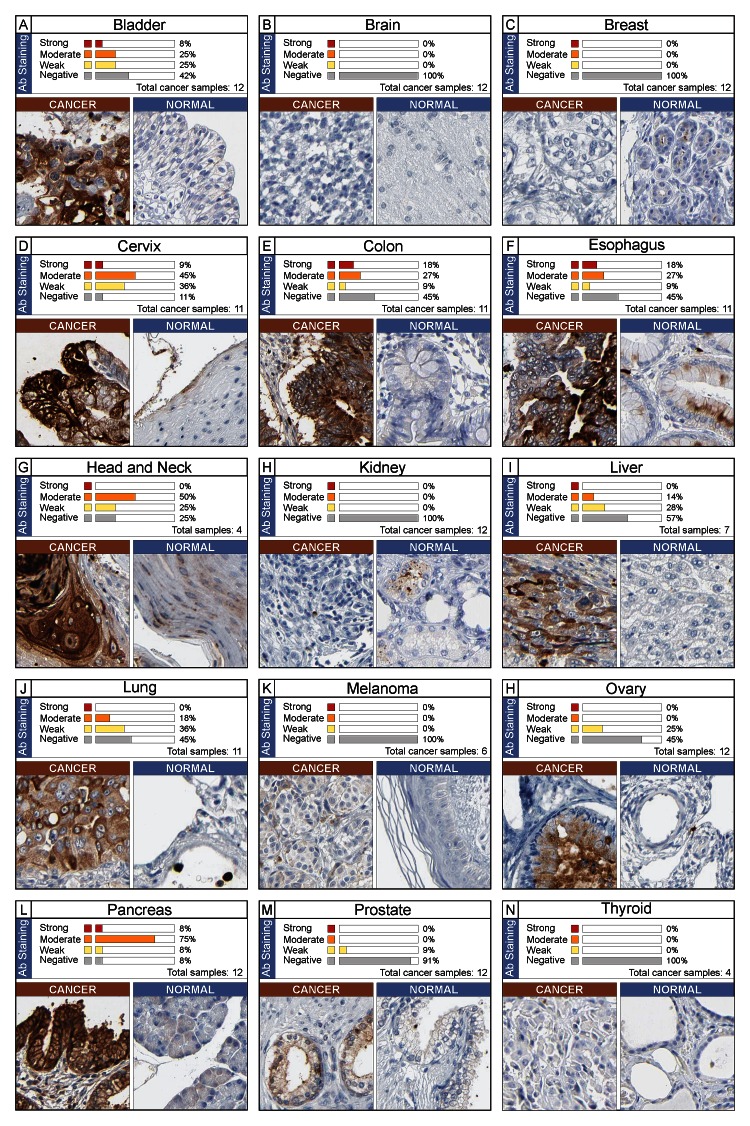
Immunohistochemistry analysis of NGAL expression in human cancer The data were obtained from the Human Protein Atlas. A single representative case for each cancer type (total 15) is shown along with its normal counterpart. Expression of NGAL in cancer sample was evaluated as strong, moderate, weak and negative immunostaining. The percentage is referred to the total cancer samples analyzed for each tumor type.

To understand if there was an association between mRNA and protein NGAL expression, an immunohistochemistry evaluation was performed by analysis of Human Protein Atlas web site. In Figure 4 immunohistochemistry analysis of NGAL in 15 solid cancer types are shown. Cancer types with negative immunostaining are not shown. The data demonstrated that concordance of NGAL at both mRNA and protein levels was obtained for the following cancer types: bladder, colorectal, liver, lung, ovarian, and pancreatic (Table 1 and Figure 4, Panels: A, E, I, J, H and L). Cervical, esophageal/stomach and head and neck cancers showed a moderate positive immunostaining (Figure 4, panel D, F and G), while mRNA expression levels were lower in cancer tissue compared to the normal counterpart (Table, 1).

Conversely, weak immunostaining (<10% of cases) or negative protein expression levels were observed in brain, breast, melanoma and prostate cancers. Accordingly, no significant differences in NGAL mRNA expression were observed between cancer and relative normal counterpart in all datasets analysed for each of these 4 cancer types (brain, breast, melanoma and prostate) (Figure 4, Panel B, C, K and M).

Although, higher mRNA levels were detected in renal and thyroid cancers than in relative normal counterparts (Table 1), a negative immunostaining was observed (Figure 4, Panel H and N). Finally, concordant negative mRNA and protein levels of NGAL were observed in the majority of haematological malignancies (data not shown).

### Clinical impact of different NGAL expression pattern

To further understand if different NGAL expression pattern may affect the clinical behaviour of several cancer types, an analysis of previously published studies was performed and is summarized in Table 3.

**Table 3:** Clinical impact of NGAL expression pattern in different cancer type according to previous studies

TUMOR TYPE	METHODS	CLINICAL IMPACT OF NGAL EXPRESSION	AUTHOR [REF.]
SOLID TUMORS			
BLADDER	CM, GZ, MS	Diagnostic marker	Roy R [24] Monier F [25]
	GZ, IHC, ELISA	Early marker of tumor progression	Monier F [26]
BRAIN	IHC, GZ, ELISA	Diagnostic marker	Smith [15]
	IHC	Positive correlation with high proliferation index in primary tumor	Barresi [28]
	IHC	Associated with poor outcome	Liu MF [27]
BREAST	IHC	Associated with poor outcome	Stoesz SP [29]
	ELISA	Positive correlation with lymphatic node metastasis	Shen ZZ [31]
	ELISA	Positive correlation with breast cancer aggressiveness	Provatopoulou X [32]
	IHC	Positive correlation with poor prognosis in primary human breast cancer	Bauer M [30]
	IHC	Positive correlation with poor outcome	Li [10]
	IHC	Positive correlation with poor outcome	Wenners [38]
CERVICAL	IHC	Positive correlation with HPV type	Syrjänen [39]
COLORECTAL	IHC, FISH	Positive correlation with tumor trasformation	Nielsen [40]
	ELISA	Prognostic utility in metastatic patients	Martì [44, 45]
	PCR, GZ	Diagnostic marker	Catalan [43]
	ELISA	Not suitable as a diagnostic marker	Fung KY [46]
	ELISA	Not useful marker of progression	McLean MH [47]
ESOPHAGEAL	GZ, IHC, WB	Positive correlation to cancer differentiation	Zhang [14]
	IHC	Positive correlation with progression	Du ZP [49]
GASTRIC	IHC, ELISA, WB	Positive correlation with poor outcome	Kubben [13]
	IHC, ELISA	Diagnostic marker and positive correlation with poor outcome	Wang HJ [60]
HEAD & NECK	ELISA	Positive correlation with poor outcome	Lin CW [61]
HCC	IHC	Positive correlation with poor outcome	Zhang Y [62]
LUNG	IHC	Positive correlation with poor outcome	Friedl A [20]
PANCREATIC	ELISA	Diagnostic marker	Moniaux N [71]
OVARIAN	IHC, ELISA, WB	Promotion of epithelial to mesenchymal transition	Lim R [19]
	PCR, IHC, ELISA	Positive correlation to cancer differentiation	Cho [65]
RENAL	IHC	Positive correlation with malignant phenotype	Barresi [76]
	ELISA	Positive correlation with poor outcome	Porta [77]
THYROID	IHC, Real time	Positive correlation with malignant phenotype	Iannetti [81]
	IHC	Diagnostic marker	Barresi [79, 80]
HEMATOLOGICAL MALIGNANCIES			
AML	RT-PCR	Positive correlation with better prognosis	Yang WC [82]
CML	RT-PCR	Positive correlation with early stage of disease and BCR-ABL positivity	Villalva C [84]

AML, Acute Myeloid Leukemia, CM, Chromatography; CML, Chronic Myelogenous Leukemia; GZ, Gel zymography; HCC, Hepatocellular Carcinoma; IHC, Immunohistochemistry. MS, Mass Spectrometry.

## DISCUSSION

Until now, NGAL has been investigated primarily as an inflammatory factor and as a marker of kidney damage [6]. However, recent studies have indicated that NGAL has potential roles in cancer development and NGAL may have pro-oncogenic or anti-oncogenic functions [22]. In fact, its oncogenic effect is related to the complex NGAL/MMP-9 [11]; while its anti-tumor effect is related to the inhibition of the pro-neoplastic factor HIF-1a, the HIF-1a-dependent VEGF and FAK [16, 17]. In the present study, computational and IHC evaluations were performed to understand whether differences in NGAL transcript or protein levels occur in different cancer types when compared with the relative normal tissues or the metastatic counterparts. The results obtained for each tumor type are discussed below in relation to previously published studies.

### Solid Tumors

 

### Bladder

The current study reveals that both mRNA transcript and protein levels were higher in bladder cancer tissues than in the normal counterparts. Accordingly, 50.5% of cases displayed NGAL transcripts above the 75^th^ percentile of the “normal” values suggesting its role as a diagnostic marker. These data are in agreement with previous investigations in which NGAL along with MMP-9 were overexpressed in urothelial bladder carcinomas suggesting their role as early diagnostic markers for this tumor type [24, 25]. Reduced protein levels of both NGAL and MMP-9 have been detected in urine samples from bladder cancer patients with clinical relapse suggesting that reduced levels of these proteins may be used as indicators of tumor progression [26].

### Brain

A significant association of MMPs and NGAL expression was detected in urine from brain cancer patients and in tumor specimens. Additionally, surgical resection of the tumor resulted in a reduction of both MMPs and NGAL in urine samples [15]. The identification of new potential molecular targets in this cancer type may be very helpful to discover new therapeutic strategies. Immunohistochemistry analysis reveals that NGAL expression is frequently up-regulated in gliomas and is associated with poor clinical outcome [27]. Barresi et al show that NGAL was overexpressed in primary high grade brain tumors and not in the metastatic cases [28]. According to the exclusion criteria designed for the purpose of our present study, no data on brain cancer were generated by computational analysis. Accordingly, NGAL protein expression was not detected through the Human Protein Atlas evaluation.

### Breast

Similar observations acquired for brain tumors were obtained for breast cancer as no data were generated by our computational analysis. The expression pattern of NGAL has been evaluated by several authors and generated conflicting results on the clinical significance of this protein in breast cancer.

A heterogeneous pattern of expression at the mRNA and protein levels was observed in breast cancer patients in a study conducted by Stoesz SP *et al* [29]. The authors also described a significant correlation between NGAL expression and other markers of poor prognosis, including estrogen and progesterone receptor-negative status and high proliferation (S-phase fraction) [29]. These studies were also confirmed by Bauer M et al [30]. Similarly, Shen ZZ et al showed that MMP-9 and MMP-9/NGAL complex expression were higher in breast cancer than in benign breast and/or normal tissues [31]. While, in the study conducted by Provatopoulou X et al. in a large series of breast cancer patients, MMP-9 and NGAL were overexpressed in cancer and this overexpression was associated with the severity of disease but no significant correlation was found for the complex formation [32].

In a transgenic mouse model of breast cancer, Berger *et al*. demonstrated that lack of NGAL in mice leads to a reduction of tumor growth. This reduction was attributed to an NGAL-dependent decrease of MMP-9 activity and to a lack of high molecular weight MMP activity [33]. Accordingly, Li *et al* have shown that NGAL expression is associated with increased metastasis and poor prognosis in breast cancer patients [10]. The results obtained by Leng *et al* in an animal model of breast cancer suggest that the suppression of NGAL function, by an inhibitory monoclonal antibody, has a great potential for breast cancer therapy, particularly by interfering with metastasis in aggressive types of breast cancer [34]. Conversely, in a previous study it was shown that NGAL overexpression promotes *in vivo* the development of lung metastasis. [35]. Our recent studies indicate that increased NGAL expression did not alter the sensitivity of the MCF-7 breast cancer cell line to the chemotherapeutic drug doxorubicin [36]. However, ectopic NGAL expression did alter the sensitivity of breast cancer cells to targeted therapy [37]. Furthermore, NGAL was found to be a predictive marker for complete response after neo-adjiuvant chemotherapy in low-risk subgroups of breast cancer patients and may be considered as an independent prognostic factor for decreased disease free survival in primary human breast cancer [38].

### Cervical cancer

A recent analysis conducted by immunohistochemistry on a set of cervical biopsy specimens from 225 women showed a close relationship between NGAL expression levels, HPV lesion grade and detection of high risk HPV types. Up-regulation of NGAL in higher grade lesions is likely to from the suppression of wild-type p53 by the HPV E6 oncoprotein. Suppression of p53 results in elimination of p53 block of NGAL transcription [39]. These data were in agreement with our IHC evaluation but not in line with our computational analysis as only 6.3% of cervical cases displayed NGAL transcripts above the 75^th^ percentile of “normal” values, while 59.4% below the 25^th^ percentile.

### Colorectal cancer

In 1996 Nielsen et a*l* analysed the role of NGAL by both immunohistochemistry and mRNA by in situ hybridization in colon cancer and in inflammatory colorectal diseases. Increased expression of NGAL was detected both in non-malignant epithelium such as diverticulitis, inflammatory bowel disease and in malignant colonic lesions. In adenocarcinomas, NGAL overexpression was observed in the transitional mucosa and in the superficial ulcerated area. On the other hand, NGAL expression was not detected in lymph node metastases from this adenocarcinoma [40]. The authors speculate that NGAL is predominantly involved in inflammatory reaction and in tumor transformation, while it does not appear to play a prominent role in metastatic process.

However, Lee et al have shown that NGAL may function as a metastasis suppressor in colon cancer cells. In their studies, they genetically manipulated highly metastatic human colon cancer cell lines, which normally express low NGAL protein levels, to overexpress NGAL. Ectopic expression of NGAL suppressed, in vivo, the liver metastasis of metastatic human colon cancer cell lines in experimentally-driven metastasis assays [18]. Hu et al examined the potential molecular mechanism of NGAL involvement in colorectal cancer. They demonstrated that NGAL overexpression altered the subcellular localization of E-cadherin and catenins, decreased E-cadherin-mediated cell to cell adhesion, enhanced cell–matrix attachment, and increased cell motility and in vitro invasion. They proposed that NGAL exerted these effects through the alteration of the subcellular localization of Rac1, one of Rho small GTPases, in an extracellular matrix-dependent manner, but not by MMP-9 [41]. Recently, Bousserouel S et al have shown, in a preclinical model of colon carcinogenesis, that NGAL is significantly upregulated only in advanced stages of tumor progression [42].

Real time PCR and zymographic analysis on visceral adipose tissue (VAT) biopsies from 11 colon cancer patients revealed increased levels of NGAL and other inflammation associated factors like osteopontin, tumor necrosis factor-α (TNF-α), and chitinase-3 like-1 compared to control subjects, suggesting their involvement in cancer development and progression [43].

Recently, Martí J et al showed the prognostic utility of NGAL mainly in metastatic CRC [44, 45]. Higher levels in colon cancer cases then controls were observed by Fung KY et al but the authors concluded that it was not a promising biomarker for the diagnosis of CRC as the sensitivity of NGAL was found to be 24% at 90% specificity [46]. Accordingly, although NGAL is still expressed by the majority of human neoplastic colorectal lesions, the author it is not a useful biomarker for determining disease progression from adenomatous to malignant colorectal neoplasia [47]. Our analysis, in line with the majority of previous studies, shows the upregulation of NGAL in adenocarcinoma tumor samples and reduced expression in metastatic samples.

### Esophageal cancer

Esophageal cancer is the eighth most common incident cancer in the world and ranks sixth among all cancers in mortality. Esophageal cancers are classified into two histological types; esophageal squamous cell carcinoma (ESCC), and adenocarcinoma, and the incidences of these types show a striking variety of geographic distribution, possibly reflecting differences in exposure to specific environmental factors. Both alcohol consumption and cigarette smoking are major risk factors for the development of ESCC [48].

Zhang *et al* performed immunohistochemistry, western blot and gelatin zymography on 81 paraffin sections including ESCC, normal mucosa, simple hyperplasia and dysplasia, and on 73 fresh specimens of ESCC to evaluate the role of NGAL in ESCC. Immunohistochemical studies revealed that ESCC have a diverse and obvious whole-cytoplasmic staining pattern for NGAL, while normal oesophageal epithelium presented a weak positive signal within a restricted cytoplasmic area. On western blot analysis, NGAL expression level was found to be significantly higher in ESCC than in normal mucosa, and positively correlated with cancer cell differentiation. No significant association was observed between NGAL expression and cell proliferation. Finally, the authors showed higher enzymatic activity of NGAL/MMP-9 complex in ESCC than in normal mucosa. These findings suggest that NGAL is involved in differentiation pathways and invasive progression of ESCC [14]. Similar data were reached by Du *et al*, [49]. Accordingly our IHC evaluations show that NGAL is overexpressed in tumor and not in normal tissue. However, different trend is observed by analyzing the transcript levels as 71.4% of cases displayed NGAL transcripts below the 25th percentile of “normal” values, while 8.6% above the 75th percentile.

Fang et al. identified a new NGALR isoform designated as NGALR-3, that results from alternative splicing. Interestingly, it was shown that the NGALR-3 isoform was overexpressed in 70% of esophageal carcinoma cases in comparison with those of normal adjacent epithelium. These findings suggest that the new NGALR-3 variant could play a more important role in esophageal carcinoma. The authors proposed that the novel NGALR-3 isoform could mediate a unilateral intracellular iron-delivery pathway which increased intracellular iron levels. This could be involved in the tumor growth of esophageal carcinomas [50].

Li EM et al studied the role of NGAL in invasion, division and proliferation of an esophageal carcinoma cell line. Their results demonstrated that the antisense blocking of NGAL transcription not only decreased effectively the activity of MMP-9 and MMP-2 secreted by SHEEC cells, but also suppressed significantly the invasion of these cells in nude mice. However, it was shown that NGAL was not apparently related with division and proliferation of SHEEC tumor cells [51]. Furthermore, the same authors, in attempt to demonstrate the regulation mechanism of NGAL overexpression in SHEEC, analyzed the structural characters of 5'-untranslated region(5'-UTR) and 3'untranslated region (3'-UTR) of NGAL. Upon cloning and DNA sequencing of 69 bp 5'-UTR and 147 bp 3'-UTR of NGAL gene they did not observe any base pair mutations [52].

### Gastric cancer

Gastric cancer (GC) is the final result of a multistep process initiated by environmental factors, including diet and *Helicobacter pylori* infection [53]. *H. pylori* infection is one of the most important risk factors for this malignancy [54, 55].

During infection, *H. pylori* synthesize siderophores, which chelate Fe^3+^ with high affinity and facilitate its transport into the pathogen [56-58]. The host cells respond to infection by increasing the secretion of NGAL that binds the bacterial siderophores and prevents their uptake into bacteria. The iron depletion results in inhibiting*H. pylori* growth [59].

NGAL and NGAL/MMP9 complex were shown to be upregulated in GC tissue (mainly in neutrophils and epithelial cells) compared to adjacent normal gastric mucosa, confirming the hypothesis that the association of NGAL with MMP9 could prevent extracellular autodegradation of the proteinase. Enhanced levels of the NGAL/MMP9 complex, but not of MMP-9 and lipocalin-2, have been related to worse clinical outcome in cancer patients and significantly associated with the classifications of Lauren and WHO, suggesting that NGAL/MMP9 complex could be considered as a novel prognostic factor for gastric cancer [13].

Wang HJ et al [60] proposed NGAL as a potential biomarker for prognosis and an ancillary diagnostic test of gastric cancer. In this study, they showed high levels of NGAL expression in 333 GC patients by immunohistochemistry. NGAL was correlated with size of tumor, Lauren's classification, lymph node metastasis, vascular invasion, distant metastasis and TNM stage. The multivariate analysis indicated that NGAL can be used as an independent prognostic factor. Serum NGAL levels were determined in blood samples from 63 healthy donors and 60 GC patients and analysed according to TNM. NGAL blood levels were higher than those of CA19-9 in TNM I patients, and higher than those of CEA and CA19-9 in TNM II. Therefore, serum NGAL has a great potential as a tumor marker for GC and could be associated with a poor prognosis [60].

According to the exclusion criteria designed for the purpose of our present study, no data on GC were generated by our analyses.

### Head and neck

Our “*in silico*” analysis showed a significant down-regulation of NGAL in head and neck cancer compared to the normal counterpart. In fact, 94.6% of head and neck cancer displayed NGAL transcripts below the 25^th^ percentile of “normal” values, while no samples showed mRNA levels of NGAL above the 75^th^ percentile. However, our IHC evaluation is in line with previous data [61] as expression of NGAL appeared to be moderate in 50% of cases and absent in normal tissue.

### Hepatocellular Carcinoma (HCC)

Zhang Y *et al* demonstrated the up-regulation of NGAL expression in HCC was significantly correlated with unfavourable clinic-pathologic features and independent poor prognostic factor for overall survival in patients [62]. In agreement, our analysis showed a significant up-regulation of NGAL mRNA levels in HCC from 2.3- to 8.6-fold changes in comparison to normal hepatic tissues in the three datasets analyzed. Similar data were obtained by IHC considerations.

### Lung cancer

At the present, few studies have been performed to evaluate the role of NGAL in lung cancer. However, Friedl A et al found high NGAL levels in lung adenocarcinoma [20]. Accordingly, in the present study we detected a constant NGAL upregulation both at mRNA and protein levels for adenocarcinoma histotype. In contrast, NGAL was not overexpressed in carcinoid lung cancer.

### Melanoma

In melanoma a substantial down-regulation of NGAL was observed only in metastatic disease versus primary tumor while no statistic differences were observed in NGAL mRNA levels between primary tumor and normal tissue. Protein expression of NGAL was not detected by IHC (Human Protein Atlas web site) (Figure 4, Panel K). To our knowledge no previous data were generated on NGAL in melanoma tissue samples.

### Ovarian cancer

Epithelial ovarian cancer is one of the most aggressive cancers diagnosed in women with high mortality rate. This cancer is usually asymptomatic and often diagnosed late in the disease process [63]. Unfortunately, there are no specific markers for early diagnosis. Lim R *et al* analysed NGAL by IHC in a total of 59 ovarian tissues including normal, benign, borderline and malignant (grades 1, 2 and 3). NGAL expression was weak or moderate in benign tissues. Both borderline and grade 1 tumors displayed the highest amount of NGAL expression with moderate to strong staining, whereas in grade 2 and 3 tumors, the extent of staining was significantly less (p < 0.01) and staining intensity was weak to moderate. Additionally, the authors analyzed, by ELISA, NGAL levels in 62 serum samples from normal individuals and ovarian cancer patients (grade 1). The NGAL concentration was 2 and 2.6-fold higher in patients with benign tumors and cancer patients (grade 1) [19]. In line with results provided by Lim*et al*, all datasets here examined showed an upregulation of NGAL in ovarian cancer samples, with a range from 3 to 6 fold. Furthermore, the analysis of both Bittner and Anglesio datasets (Ref. in Table 2) revealed a significant reduction of NGAL mRNA levels in metastatic disease (see Table 2). In the same study, Lim and collaborators analyzed NGAL in ovarian cancer cell lines treated with epidermal growth factor (EGF) indicating that NGAL expression was downregulated in ovarian cancer cell lines undergoing the epithelial to mesenchymal transition (EMT) induced by EGF. Downregulation of NGAL expression correlated with the upregulation of vimentin, enhanced cell dispersion and downregulation of E-cadherin expression, some of the hallmarks of EMT. These data indicate that NGAL may be a good marker to monitor transformation of benign lesions to premalignant and malignant ovarian tumors and that the molecule may be involved in the progression of epithelial ovarian malignancies [19].

NGAL, as well as other proteins, was shown to be regulated in ovarian cancer cell lines by 17 beta-estradiolestrogen [64]. More recently Cho et al. described the upregulation of NGAL in a panel of 54 ovarian cancers, 15 borderline and 53 benign ovarian tumors, and 90 healthy controls by real time PCR and immunohystochemical analysis. NGAL levels were significantly higher in ovarian tissues and particularly in well-differentiated tumors. Similar results were obtained by analyzing NGAL serum levels from ovarian cancer patients showing highest levels in differentiated cancer [65]. Notably, according to previous data, the results of the present study show that 91,2% of ovarian cases displayed NGAL transcripts above the 75^th^ percentile of the “normal” values, while 0.4% below the 25^th^ percentile. Similar trend was obtained for the IHC analyses. While transcript levels of NGAL were significantly lower in metastatic setting compared to primary tumor.

### Pancreatic cancer

Pancreatic cancer (PaCa) is the fifth leading cause of cancer death in both men and women [66]. Early detection of this disease is not possible in spite of significant diagnostic tools. The effective therapy, surgery, is limited to about 25% of the cases and often is unable to prevent cancer recurrence in these patients [67]. Much remains to be understood about the natural course and biology of this disease.

Using microarray analysis, many laboratories have reported the differential expression of several novel genes, including that of NGAL, associated with the progression of pancreatic cancer [68-70]. Moniaux *et al* detected NGAL levels by immunohistochemistry on tissue samples from normal patients, pancreatitis, and pancreatic adenocarcinoma patients. Their results revealed higher levels in pancreatic adenocarcinoma than in normal and pancreatitis samples [71]. These findings are in agreement with the results of the present study in which we observe a constant upregulation of NGAL at both mRNA and protein expression levels in pancreatic adenocarcinoma samples vs normal tissues. Moniaux *et al* also evaluated NGAL levels in pancreatic cancer cell lines with varying grades of differentiation documenting a positivity for NGAL expression in both well and moderately differentiated cells. In contrast, NGAL expression was uniformly negative in poorly differentiated adenocarcinoma. Further, they examined NGAL levels in serum samples. They used ELISA to detect NGAL. The authors concluded that NGAL was fairly accurate in distinguishing between pancreatic cancers and non-cancer cases. In conclusion, NGAL is highly expressed in early pancreatic dysplastic lesions, suggesting a possible role as an early diagnostic marker for pancreatic cancer [71].

NGAL is also detected in bile and may be useful as a novel biomarker to distinguish benign from malignant biliary obstruction [72].

The biological roles of NGAL in pancreatic adenocarcinoma have been studied both *in vitro* and *in vivo* by Tong *et al* [17]. The authors transfected PaCa cells with NGAL and demonstrated no effects on cell viability or sensitivity to chemotherapy, but decreases in cell adhesion, invasion and angiogenesis was observed both *in vitro* and *in vivo*. The negative effects on tumor progression and metastasis were due to alteration of FAK phosphorylation and decrease of VEGF production [17]. We can gather from this study that modulation of NGAL activity could control PaCa angiogenesis and metastasis.

### Prostate cancer

According to the exclusion criteria designed for the purpose of our present study, no differences were identified between prostate cancer tissue and the normal counterparts. Similarly, IHC evaluation did not display any differences between normal and tumor. While, significant differences were observed in metastatic disease of prostate cancer when compared with primary tumors as NGAL transcripts were lower in metastatic setting compared to primary tumor. However, in vitro studies showed that NGAL plays a significant role in the progression of prostate cancer by regulating MMP2 and MMP-9 [73]. Therefore further studies are needed to better clarify the role of NGAL in prostate cancer, as until now conflicting results have been generated between “in silico” and “in vitro” data.

### Renal tumor

The incidence of renal cell carcinoma (RCC) is growing [74]. Most of the patients initially diagnosed with localized disease are cured by surgery, but over 30% of them die from relapse. The molecular basis of a great diversity in clinical behavior of RCC is still unclear and makes it a target to investigate the nature of these heterogeneities [75].

A recent immunohistochemical study on a set of 30 surgically-resected renal tumors revealed that NGAL is expressed in several histotypes of renal tumors especially in papillary and chromphobe histotypes. NGAL expression is highest in the higher histological grade of papillary and clear cell RCC and in its peritoneal metastasis [76].The authors suggested that the upregualtion of NGAL in the above-mentioned tumor histotypes could be related to an increased requirement of iron uptake and could justify the use of iron chelators for renal cancer therapies. In agreement, our analysis revealed an upregulation of NGAL in RCC. Conversely, IHC evaluation did not show any immunostaining for NGAL. It was further shown that NGAL, as detected by ELISA, had predict value for progression free survival in RCC patients treated with sunitinib malate [77]. While, most recent data conducted by Di Carlo does not reach any conclusive results on the usefulness of NGAL as a diagnostic marker [78].

### Thyroid cancer

Our data show that transcript levels of NGAL are higher in tumor thyroid cancer (papillary carcinoma) when compared with normal counterpart. In fact, 83.7% of case displayed NGAL transcripts above the 75^th^ percentile of “normal” values, while 6.1% of cases showed mRNA levels of NGAL below the 25^th^ percentile (Figure 3, Panel K). In contrast, no protein expression was detect by IHC (Human Protein Atlas web site). According to mRNA expression data, previous studies showed that NGAL is overexpressed in thyroid cancer [79-81].

### Hematological malignancies

Different then for solid tumor, NGAL expression in hematological malignancies displays a very homogeneous behavior: in fact, all the datasets that reach the levels of significance p<0.01 exhibit an uniform downregulation of NGAL compared to controls. Datasets analyzed included myeloma and leukemias (ALL, acute lymphoblastic leukemia; AML, acute myeloid leukemia, CLL; chronic lymphocytic leukemia). Accordingly, recent data show that highest mRNA NGAL levels were associated with better prognosis in AML [82]. According to the exclusion criteria designed for the purpose of the present study, no data on chronic myeloid leukemia (CML) were generated by computational analysis. However, NGAL was identified in an expression profiling study on CML cells. In this work, the authors analysed gene expression profiles of cancer cells from 27 patients using a cDNA microarray. Among the 150 genes up-regulated, they observed an increase of NGAL mRNA levels in CML patients [83]. Subsequently, Villalva C et al have performed RT-PCR for NGAL expression in a large cohort of CML patients. Their results indicate that NGAL is expressed in parallel with the BCR-ABL oncoprotein at the early stage of leukemia process and it is secreted at high levels in these patients. The authors have excluded the possibility that the large increase of NGAL expression in CML patients at diagnosis resulted from the presence of circulating myelocytes in blood, which constitutively secrete NGAL protein during maturation [84]. Leng et al have proposed two activities associated with NGAL in a mouse model of CML tumorigenesis. On the one hand, NGAL induces apoptosis of normal hematopoietic cells resulting in the replacement of these with leukemic cells and on the other NGAL facilitates tissue invasion through stabilization of MMP-9 activity [85].

## CONCLUSION

Our computational analysis suggest an active role of NGAL in early stages of tumor development, reason that many authors proposed NGAL as a diagnostic and prognostic marker. While a decrease of NGAL expression in metastatic samples was detected when compared to matched primary tumors. This observation suggests a common inactivation pathway of NGAL gene during distant tumor dissemination and leads us to venture the hypothesis that NGAL could play a protective role in metastatic development.

In particular, the tumors showing higher NGAL expression (expression levels greater than the 75^th^ percentile of “normal” samples) were: ovarian (91.2%), thyroid (83.7%), liver (68.8%), colon (66.3%), kidney (64.7%), lung (63.1%), pancreas (60.2%) and bladder (50.5%). While, the percentages of tumor cases showing NGAL transcripts below the 25^th^ percentile of “normal” values were almost 100% of all hematological malignancies and 94.6% of head and neck cancer, 71.4% of esophagus cancer and 59.4% of cervical carcinoma. These data suggest that NGAL is a candidate marker for tumor growth in a fraction of solid tumor and a favorable prognostic factor for the remaining cancer types showing lower levels of NGAL transcript levels.

The analysis of previous studies revealed a lack of information about NGAL correlation with metastasis and almost completely consistent with the bionformatic data. The present study deepens the knowledge of the molecular mechanisms sustaining NGAL expression in tumor cells and its effects on cancer metastatic behavior. Further investigations are required to elucidate the function of NGAL in tumor development and metastatic processes.

## MATERIAL AND METHODS

ONCOMINE software (https://www.oncomine.com) was used (December 2013) to compare mRNA expression levels between normal tissue versus tumor and primary tumor versus metastatic, and in both cases evaluated on biopsy samples. Statistical analysis of the differences in NGAL mRNA expression between the abovementioned sets of samples was accomplished through use of ONCOMINE algorithms. Only datasets generated by Affymetrix U133 platform were considered for the present analysis. Of note, this platform used the “212531_at” NGAL probe set. Datasets showing different expression analysis between normal and tumor tissues with statistical significance less than 0.01 (by t-test) and fold change ≤ -2 or ≥ 2 were included; while, those showing a differential expression between primary tumor and metastasis with a statistic significance less than 0.05 (by t-test) and fold change ≤ -1.5 or ≥ 1.5 were considered. According to these criteria, 38 datasets were used for the purpose of the study. Of these, 29 were used for the comparative analysis between normal and tumor tissues [86-111]; while, 9 were used to analyze the differences between primary tumor and metastasis [112-118]. Fold change was calculated to evaluate the changes in gene expression between the groups of samples included in this analysis. Properties of the datasets are presented in Tables 1 and 2.

To assess the distribution of NGAL transcript levels among cancer types and normal samples, mRNA expression levels from Oncomine analysis were normalized using an housekeeping gene in each dataset and then merged together. The choice of the housekeeping gene was made on the basis of the homogenous mRNA levels distribution. When more than one probset was available, the mean value of the same housekeeping gene was used for the normalization. To define the “normal range” of expression we calculated the 25th and the 75th percentile of NGAL transcript levels in normal samples for each tumor type. Accordingly, the percentage of tumor samples showing NGAL levels outside the defined “normal range” was calculated.

NGAL protein expression was performed by analyzing the web site of Human Protein Atlas (http://www.proteinatlas.org/). As indicate in the web site, expression of NGAL for each tumor type was evaluated as strong, moderate, weak and negative immunostaining. The Sigma-Aldrich HPA002695 NGAL antibody was employed for this analysis.
